# Carnosic acid inhibits the proliferation and migration capacity of human colorectal cancer cells

**DOI:** 10.3892/or.2012.1630

**Published:** 2012-01-11

**Authors:** M.V. BARNI, M.J. CARLINI, E.G. CAFFERATA, L. PURICELLI, S. MORENO

**Affiliations:** 1 Foundation Institute Leloir, IIBBA-CONICET, CABA 1405 Buenos Aires; 2 Institute of Oncology Ángel H. Roffo, CABA 1417 Buenos Aires, Argentina

**Keywords:** carnosic acid, colorectal cancer, migration, antitumor, cyclooxygenase-2

## Abstract

Colorectal cancer (CRC) is the third most common malignant neoplasm worldwide. The objective of this study was to examine whether carnosic acid (CA), the main antioxidant compound of *Rosmarinus officinalis* L., would inhibit the cell viability of three CRC cell lines: Caco-2, HT29 and LoVo in a dose-dependent manner, with IC_50_ values in the range of 24–96 μM. CA induced cell death by apoptosis in Caco-2 line after 24 h of treatment and inhibited cell adhesion and migration, possibly by reducing the activity of secreted proteases such as urokinase plasminogen activator (uPA) and metalloproteinases (MMPs). These effects may be associated through a mechanism involving the inhibition of the COX-2 pathway, because we have determined that CA downregulates the expression of COX-2 in Caco-2 cells at both the mRNA and protein levels. Therefore, CA modulates different targets involved in the development of CRC. These findings indicate that carnosic acid may have anticancer activity and may be useful as a novel chemotherapeutic agent.

## Introduction

Many epidemiological studies show a relationship between diet and the incidence of colorectal cancer (CRC). In particular, it is known that approximately 60–75% of all sporadic CRC cases are directly influenced by diet (1). For this reason, chemoprevention presents a major strategy for the medical management of CRC and several natural compounds are being investigated by many researchers as possible inhibitory agents for CRC initiation and progression (2–4).

It is widely known that tumor formation is a complex, multistep process involving the accumulation of genetic lesions in genes that regulate the pathways of cell proliferation, adhesion, differentiation and death required for normal development. In order to invade, epithelial cancer cells need to penetrate through the basement membrane and to disorganize the extracellular matrix (ECM). In this context, proteases play a key role since they can either degrade or process the ECM components and thereby support cancer cell invasion (5). It is well known that tumor cells produce higher amounts of proteolytic enzymes than their normal counterparts. In particular, the matrix metalloproteinase (MMP) MMP-2 and MMP-9 and the urokinase plasminogen activator (uPA) are responsible for the degradation of several ECM components and play important roles in the process of human colon cancer invasion and metastasis (6).

Cyclooxygenase (COX) enzymes catalyze the enzymatic conversion of arachidonic acid to prostaglandins (PG). Constitutive COX-1 is responsible of physiological PG levels, whereas inducible COX-2 is expressed upon stimulation and accounts for high PG levels. COX-2 is overexpressed in a number of human and murine cell lines and tumors (7). In relation to cancer, PG are able to promote tumor growth by inducing cell proliferation and/or inhibiting the apoptosis of tumor cells, stimulating the release of MMPs and/or tumor cell migration, finally favoring metastatic dissemination (8). For instance, it was observed that COX-2 overexpression in Caco-2 human colon cancer cells, stimulates cell migration and invasion, associated with higher expression of several proteases of the MMP family (9).

Plant-derived polyphenols are a large group of naturally occurring antioxidants. Epidemiological studies have suggested that a polyphenol-rich intake from fruits and vegetables is associated with decreased risk of different diseases, including cancer (10). This may be due to the fact that polyphenols are able to act as negative regulators of inflammation or because they may serve as signaling agents themselves (11). In particular, *Rosmarinus officinalis* L. (rosemary), the most popular spice of the Lamiaceae family, is a rich source of polyphenols as carnosic acid (CA), carnosol (COH) and rosmarinic acid (RA). It was reported that CA has many pharmacological activities (12,13), as inhibiting the proliferation of the human promyelocytic leukemia cells HL-60 and U937 (14–16). Furthermore, CA has been shown to have anti-inflammatory properties, to reduce the expression of cytokine-induced adhesion molecules, to block the adhesion of monocytes to endothelial cells (17), and to prevent the migration of human aortic smooth muscle cells by suppressing the expression of MMPs (18).

Previously, we have studied the antioxidant and antibacterial activities of the more conspicuous non-volatile polyphenols isolated from *Rosmarinus officinalis* L., as CA and RA, employing different *in vitro* and *in vivo* approaches (19–21).

In the present study, we demonstrated the antitumoral action of CA on three human colon cancer lines with different genetic background: Caco-2 (p53^m^), LoVo (p53^wt^) y HT29 (p53^wt^). We found that CA reduces cell viability by inducing apoptosis in Caco-2 cell line, and inhibits cell migration ability, probably due to the inhibition of uPA and MMP-9 protease activities. In addition, CA inhibited COX-2, at mRNA and protein levels. These findings suggest that CA may provide a new therapeutic strategy useful for the treatment of CRC disease.

## Materials and methods

### Reagents and rosemary plant compounds

**Figure 1:**
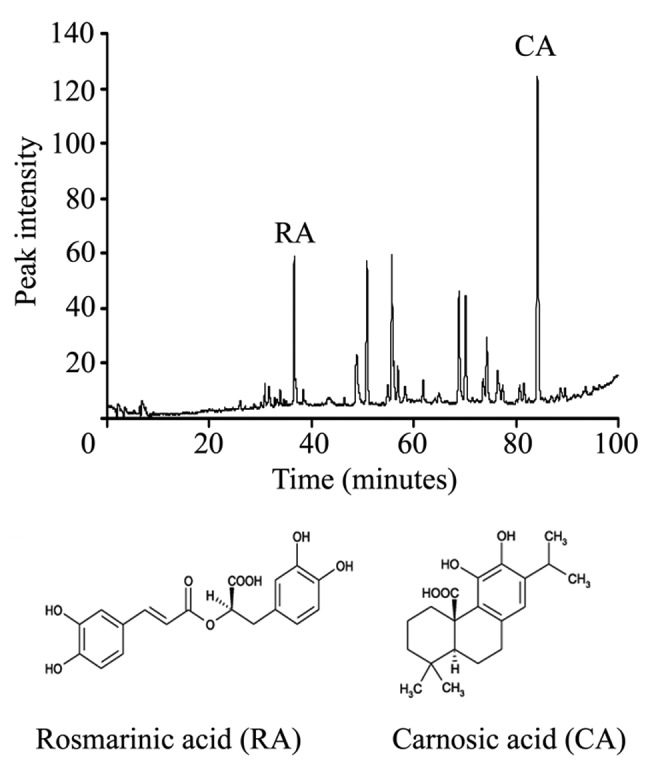
Chromatographic profile of the extract of *R. officinalis* L. determined by HPLC (A). Molecular structures of RA (left) and CA (right) (B).

Carnosic acid (CA) and rosmarinic acid (RA) were purchased from Alexis Biochemicals (USA). *R. officinalis* L. extract (RE) was obtained from dried leaves by ethanol extraction and the identification of RE compounds was performed by HPLC as previously described (19). Stock solutions were prepared in ethanol 100% and stored at −20°C. Fig. 1A shows that the RE contained two main peaks corresponding to 10% CA and 3% RA, and Fig. 1B shows the structures of RA and CA.

### Cell culture

Human colon carcinoma cell lines, Caco-2, HT29 and LoVo were grown in DMEM (Gibco/Invitrogen, USA) with HyQ Ham’s/F-12 (HyClone, Thermo Scientific, USA) and supplemented with 10% fetal bovine serum (Internegocios, Argentina), 100 μg/ml streptomycin and 100 U/ml penicillin-G at 37°C in a humidified 5% CO_2_-air atmosphere. Cells were grown to 70% confluence and subcultured 2–3 times a week using 0.25% trypsin-EDTA (Gibco/Invitrogen).

### Cell viability assay

Cells (1×10^4^) were seeded in 96-well microplates in complete medium. After 48 h, cells were washed twice with PBS and treated with RE, RA and CA (concentration range from 0 to 388 μM) in complete medium for 24 h. Cell viability was assessed by the CellTiter 96 Aqueous Non-Radioactive Cell Proliferation Assay (Promega, Madison, WI) following the manufacturer’s recommendations and monitored by absorbance at 595 nm in a microtiter plate reader (Beckman-Coulter DTX880 Multimode Detector). IC_50_ was produced using Microcal Origin 6.0 Professional analysis software.

### Annexin-V-Cy3/6-carboxyfluorescein diacetate staining

Phosphatidylserine translocation from the inner to the outer leaflet of the plasma membrane is one of the early apoptotic features. Cell surface phosphatidylserine was detected by phosphatidylserine-binding protein Annexin-V conjugated with Cy3.18 using the Annexin-V-Cy3 apoptosis detection kit (Sigma-Aldrich, USA) (22). Briefly, Caco-2 cells (3×10^4^) were cultured in glass coverslips on 24-well microplates. After 24 h cells were washed with PBS and treated or not with CA (IC_50_ dose) for additional 24 h. Then, cells were washed with PBS and incubated with 50 μl of double label staining solution (containing 1 mg/ml AnnCy3 and 100 mM 6-carboxyfluorescein diacetate) for 10 min at room temperature in the dark. Cells were then washed three times with 50-μl binding buffer followed by immediate observation using a confocal and fluorescence microscope (LSM 5 Pascal, Axioplan 2 Imaging). The combination of 6-carboxyfluorescein diacetate (6-CFDA) with Cy3-conjugated Annexin-V allowed the differentiation between live (green), necrotic (red), and apoptotic cells (red and green).

### DAPI nuclear staining

Caco-2 cells were cultured on 24-well microplates (3×10^4^/well) for 24 h. Cells were then washed with PBS and treated or not with CA (concentration range from 0 to 388 μM). Cells were washed with PBS twice and fixed with 4% formaldehyde in PBS for 1 h at room temperature, washed twice with H_2_O and then kept in PBS for 30 min at room temperature. Finally, cells were stained with 300 μl of 30 nM DAPI (Molecular Probes, USA) in PBS for 5 min in the dark. Cells were observed for nuclear condensation/fragmentation indicative of apoptosis with an inverted and fluorescence microscope (Axiovert 135M, Carl Zeiss) and photographed using a high resolution camera. Three fields were photographed and the percentage of total apoptotic cells compared to the total number of cells (100–300 cells) was determined. Each condition was assayed in triplicate.

### Adhesion assay

Cell adhesion assay was performed with modifications of previously described methods (5,23). Ninety-six-well microplates were coated with matrix proteins (40 μg/ml type I collagen or 2 μg/cm^2^ fibronectin in PBS) at room temperature for 1 h, washed twice with PBS and blocked with 100 μl of 1% BSA in PBS for 2 h at 37°C. BSA-coated wells were used as negative controls. Wells were washed twice with 100 μl PBS. Cells (2.5×10^4^ cells/100 μl) were suspended in culture medium with CA (concentration range from 0 to 388 μM) and added to each well. After 1-h incubation at 37°C, cells were inspectioned using a microscope, washed gently with PBS, fixed with 50 μl of methanol, washed twice with H_2_O, stained with 2% crystal violet for 10 min and washed twice with water. Finally, 50 μl of 10% methanol and 5% glacial acetic acid solution was added to each well and the optical density at 595 nm was measured in a microplate reader (Beckman Coulter DTX880 Multimode Detector). Adhesion of CA-treated cells was related to the control set at 100%.

In another set of experiments, 24-h-CA pre-treated cells (same concentration range) were washed, suspended in culture medium and then seeded onto the coated wells. Then, the adhesion assay was performed as described above.

The morphology of control and pre-treated cells with CA at IC_50_ were photographed using an inverted microscope (Axiovert 135M, Carl Zeiss).

### Migration assay

Caco-2 confluent monolayers were manually scratched with a pipette tip to create scratches in the center of the dishes. Detached cells were removed by washing the cells twice with PBS and serum-free medium, with or without CA (concentration range from 0 to 388 μM), was added to each dish. Each treatment was performed in triplicate. Four images per well were taken immediately after adding treatments and 24 h later using an inverted microscope (Axiovert 135M, Carl Zeiss). Unpopulated areas were analyzed using Image-Pro Plus analysis software by measuring unpopulated area at 0 and 24 h and cell advancement area was derived for each treatment. Data were expressed as a percentage from untreated control cells (set as 100%).

### Urokinase plasminogen activator activity

A radial caseinolysis assay (24), using plasminogen-rich (2 μg/ml) casein-agarose plates, was employed to quantitate uPA activity in the conditioned media of control or CA-treated cells obtained as described above. Radial caseinolysis of conditioned media were photographed with a digital densitometer and quantified using Image-Pro Plus 5.1 analysis software. uPA activities were referenced to a standard urokinase curve (0.1–50 UI/ml), normalized to the original protein concentration content by Bradford assay and uPA activities were expressed as a percentage from untreated control cells (set as 100%).

### Gelatin zymography

Caco-2 subconfluent (70%) 35-mm plates were washed with PBS to remove growth factors, and the cells were fed with 1-ml serum-free medium with or without CA (concentration range from 0 to 388 μM). After 24 h, the conditioned medium was collected, centrifuged to remove cellular debris, and stored at −20°C until use. MMP enzymatic activity was determined on substrate-impregnated gels. Briefly, 10 μl of conditioned mediums were separated on 9% SDS polyacrylamide gels containing 1 mg/ml of gelatin (Sigma-Aldrich), under non-reducing conditions. After electrophoresis, gels were washed for 20 min in 2.5% Triton X-100 and incubated for 24 h at 37°C in 50 mM Tris-HCl (pH 7.4), 200 mM NaCl, 5 mM CaCl_2_ and 0.02% Triton. Gels were fixed and stained with 0.5% Coomassie brilliant blue G-250 (Bio-Rad Laboratories, Richmond, CA) in methanol/acetic acid/H_2_O. Activity bands were visualized by negative staining. Gelatinolytic bands were measured with a digital densitometer and quantified using Image-Pro Plus 5.1 analysis software. Data were expressed as arbitrary units and normalized regarding control cell values (set as 100%) and the original cell lysate protein content, determined by Bradford assay.

### RNA extraction and reverse transcriptase-polymerase chain reaction (RT-PCR)

Caco-2 cells were cultured on 6-well microplates (5×10^4^/well) for 24 h. Cells were then washed with PBS and treated or not with CA (concentration range from 0 to 388 μM) for 24 h. Total RNA was extracted using TRIzol reagent (Invitrogen) according to the manufacturer’s instructions. For RT-PCR, cDNA was made from total RNA using random primers and the M-MLV Reverse Transcriptase kit (Promega) according to the manufacturer’s protocol. PCR amplification was performed with the following primers (forward and reverse): 5′-GAGCGTCAGTATCAACTGCG-3′ and 5′-ATTGGAACTGGACACCGAAC-3 for COX-1, 5′-TTC AAATGAGATTGTGGGAAAATTGCT-3′ and 5′-AGATCA TCTCTGCCTGAGTATCTT-3′ for COX-2; 5′-CCACCCAT GGCAAATTCCATGGCA-3′ and 5′-TCTAGACGGCAGGT CAGGTCCAC-3′ for GAPDH. PCR amplification was carried out with 30 ng of cDNA template in a volume of 50 μl reaction mixture containing 5 μl reaction buffer (10x), 0.25 mM dNTPs (Promega), 0.25 μM of each primer, and 1 U of Pfu DNA polymerase (Institute Leloir, Argentina). PCR was performed with a thermal cycler (GeneAmp PCR System 9600, Perkin-Elmer) under the following conditions: 94°C/1 min; 60°C/1 min, 72°C/1 min for 35 cycles. Amplified cDNAs were electrophoresed on 1% agarose gel stained with ethidium bromide. Results were quantified with Scion Image analysis software.

### Western blot analysis

Caco-2 cells were cultured on 6-well microplates (5×10^4^/well) for 24 h. Cells were then washed with PBS and treated or not with CA (concentration range from 0 to 388 μM) for 24 h. Then, the cells were washed with PBS and lysed with RIPA buffer containing 150 mM NaCl, 1% NP-40, 50 mM Tris-HCl (pH 8.0), 1 mM EDTA, 0.5% deoxycholate, 100 μg of phenylmethylsulfonyl fluoride for 30 min on ice, centrifuged at 13000 rpm at 4ºC for 15 min, and equal amounts (50 μg) of the supernatant proteins were used in western blots with rabbit polyclonal COX-2 (1:1000) and rabbit polyclonal β-actin (1:1000, Santa Cruz Biotechology) as loading control. Proteins were separated by 10% SDS-PAGE and transferred onto nitrocellulose (Amersham Hybond-P, GE Healthcare). The membranes were blocked for non-specific binding for 1 h in 5% milk (w/v) diluted in PBS Tween-20. The blots were then incubated overnight with primary antibodies. Subsequently, the blots were washed and ECL anti-rabbit IgG, horseradish peroxidase-linked whole antibody from donkey (1:5000) (GE Healthcare UK). After further washing, the blots were subjected to enhanced chemiluminescence detection system (ECL Plus, GE Healthcare) reagent and monitored with Molecular Dynamics Storm B40 scanner. Results were quantified with Scion Image analysis software.

### Statistical analysis

Results are expressed as means ± SD. Differences among groups were analyzed by Student’s t-test and ANOVA test. Values of p≤0.05 were considered statistically significant.

## Results

### Effect of CA the main bioactive of R. officinalis on the viability of three CRC cell lines

Previously we studied the antiproliferative activity of several rosemary extracts (RE) using the microplate colorimetric MTS assay employing human colon cancer cells (Biocell).

**Figure 2:**
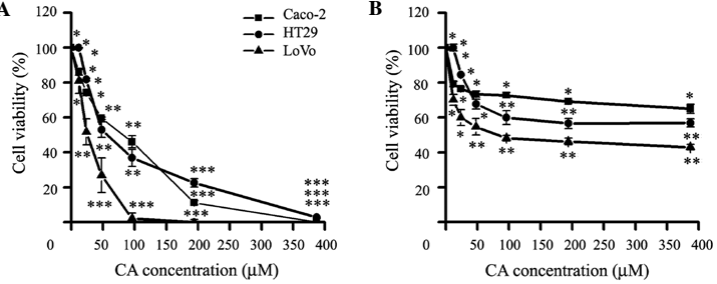
Effect of CA (A), RA (B) on cell viability of Caco-2, HT29 and LoVo cell lines. Cells were exposed to CA and RA (0 at 388 μM) for 24 h and cell viability was measure by MTS assay. The values represent means ± SD of three cultures from triplicate-independent experiments. (^*^P<0.05, ^**^P<0.01, ^***^P<0.001 vs. control).

We evaluated whether CA was able to modulate the *in vitro* growth of three human CRC cell lines with similar population doubling time: Caco-2, HT29 and LoVo cells. Later, we tested the two main constituents of the RE, the CA and RA (Fig. 1), CA was the main bioactive compound inhibiting the viability of the three CCR cell lines, while RA showed a significant activity at high concentrations (Fig. 2).

As shown in Table I, CA was able to inhibit cell viability at different degrees after 24 h. CA significantly inhibited growth in a dose-dependent manner IC_50_ (μM): Caco-2, 92.1±6.4; HT29, 48.5±8.2 and LoVo, 26.4±2.7. Therefore, CA compound was selected for the subsequent studies.

### Induction of apoptosis by CA

It is well known that cell viability of tumor cell populations is determined by the balance between proliferation and death; here, we studied the effect of CA on survival of Caco-2 cells using two different approaches: Annexin-V and DAPI staining.

**Figure 3:**
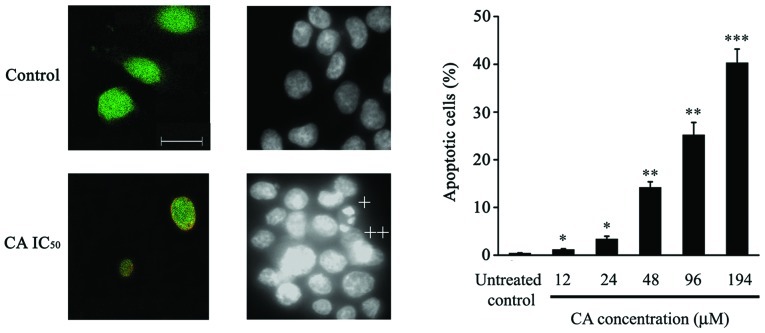
Effect of CA on Caco-2 apoptotic cell death. (A) CA induces phosphatidylserine translocation, one of the early apoptotic features, in Caco-2 cells. Cells were cultured in absence (control) and presence (CA IC_50_) for 24 h and exposed to double-stained with 6-CFDA and Annexin-V-Cy3 and photographed with confocal and fluorescence microscopy. Similar results were obtained from two additional separated experiments. Fluorescence microscopic images of Caco-2 cells treated with CA at IC_50_ for 24 h and stained DAPI dye. (B) CA induces signs of late apoptotic features such as apoptotic bodies (+) and condensation of the nuclear material (++). Scale bar, 20 μm. (C) The percentage of apoptotic cells, stained as indicated in B, was recorded. The values represent mean ± SD of three independents experiments. (^*^P<0.05, ^**^P<0.01, ^***^P<0.001 vs. control).

As depicted in Fig. 3A, intact control cells only showed 6-CFDA staining, while 24-h-CA-treated cells (IC_50_) showed increased numbers of cells stained with both Annexin-V-Cy3 and 6-CFDA (Fig. 3A), suggesting that these cells were undergoing apoptotic cell death.

DAPI staining assessment showed that CA treatment induced typical apoptotic features in Caco-2 cells, such as chromatin condensation, loss of normal nuclear architecture and apoptotic bodies (Fig. 3B). Furthermore, a significant increase in the percentage of apoptotic cells dependent on CA dose was observed (Fig. 3C).

### Inhibition of cell adhesion by CA

**Figure 4:**
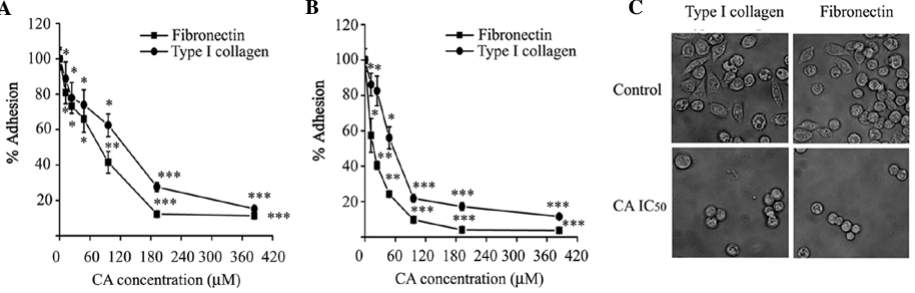
Inhibition of adhesion cells by CA treatment. (A) Caco-2 cells were cultured in 96-well multiplates, previously coated with type I collagen or fibronectin, in the presence or absence of CA (0–388 μM) for 1 h. Then, cells were stained with 2% crystal violet and the optical density at 595 nm was measured. (B) Cells were pre-incubated with CA for 24 h, washed and cultured as described above. (C) Fluorescence-microscope images of untreated and pre-treated cells with CA at IC_50_ for 24 h were taken after 1 h of incubation onto the coated wells with type I collagen (left) and fibronectin (right). The values represent means ± SD of six cultures from duplicate experiments. (^*^P<0.05, ^**^P<0.01, ^***^P<0.001 vs. control). Scale bar, 20 μm.

Adhesion of Caco-2 cells was measured on immobilized ECM proteins (type I collagen and fibronectin) in the presence of CA. As shown in Fig. 4A, adhesion of Caco-2 cells to type I collagen and fibronectin was significantly impaired in the presence of CA, compared with adhesion to BSA used as control, when the assay was performed for 1 h. The effect of CA on cell adhesion was dose-dependent, the doses were able to inhibit adhesion by half of control values (124±6.54 μM) on type I collagen and 77.57±5.22 μM) on fibronectin.

In a different approach, cells were pre-treated with CA for 24 h prior to the adhesion assay. As expected, CA-pretreated cells showed 2- to 4-fold lower adhesion to the substrates in this condition. In addition, CA pre-treatment completely and significantly inhibited the spreading of Caco-2 cells within 1 h of incubation on type I collagen and fibronectin surfaces compared to untreated control cells (Fig. 4C). These results indicate that CA is able to impair the adhesion of Caco-2 cells to ECM proteins.

### Inhibition of cell migration by CA

**Figure 5:**
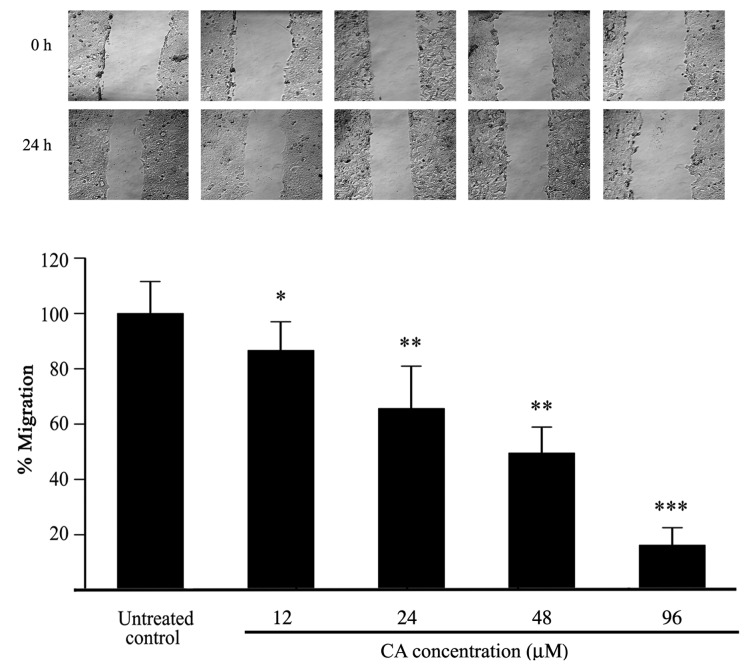
Effect of CA on the migration of Caco-2 cells. (A) Confluent cell monolayers were wounded with a pipet tip and incubated without or with CA (0 at 388 μM) for 24 h. Wound were monitored and photographed at 0 and 24 h of incubation with a phase-contrast microscope. The percentage of migration is shown in B. The values represent mean ± SD of quadruplicate cultures from duplicate experiments. (^*^P<0.05, ^**^P<0.01, ^***^P<0.001 vs. control).

To evaluate the effects of CA on the migration ability of Caco-2 cells, a wound healing assay was performed on confluent cell monolayers. Cells were treated with increasing concentrations of CA for 24 h. Cell migration was clearly inhibited by CA after of treatment in comparison with untreated controls as observed by optical microscopy (Fig. 5A). As shown in Fig. 5B, CA significantly inhibited migration of Caco-2 cells dose-dependently, with 48 μM CA inhibiting migration by 50%, although significant inhibition of migration was already evident at 24 μM which had little effect on cell viability.

### Inhibition of secreted protease activity by CA

**Figure 6:**
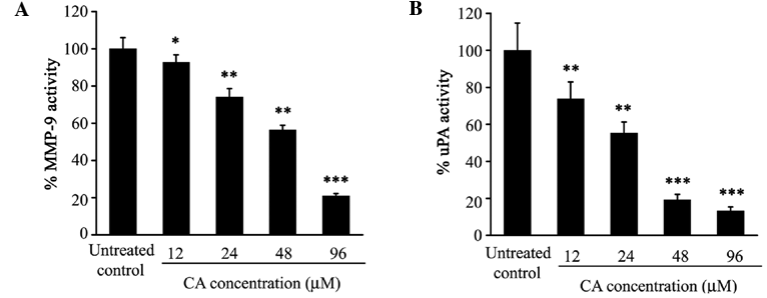
Effect of CA on extracellular matrix components activity of Caco-2 cells. Cells were incubated without or with CA (0 at 96 μM) for 48 h. Samples of conditioned medium were seeded in agarose gel with casein and plasminogen and then were incubated in a humidified chamber at 37°C for 48 h. uPA activities were quantified by radial caseinolisis and determined the percentage of inhibition of uPA activity. The values represent mean ± SD of three experiments (^**^P<0.01, ^***^P<0.001 vs. control).

The effect of CA on secreted uPA activity was analysed by radial caseinolysis assay. CA treatment significantly inhibited this activity in the conditioned media, since 8 μg/ml (24 μM) of CA was able to reduce the uPA activity approximately by a half (Fig. 6). Preliminary results using gelatin zymography showed that 48 μM of CA inhibited approximately 50 and 80% the MMP-9 and MMP-2 activities, respectively.

### Inhibition of COX-2 expression by CA

**Figure 7:**
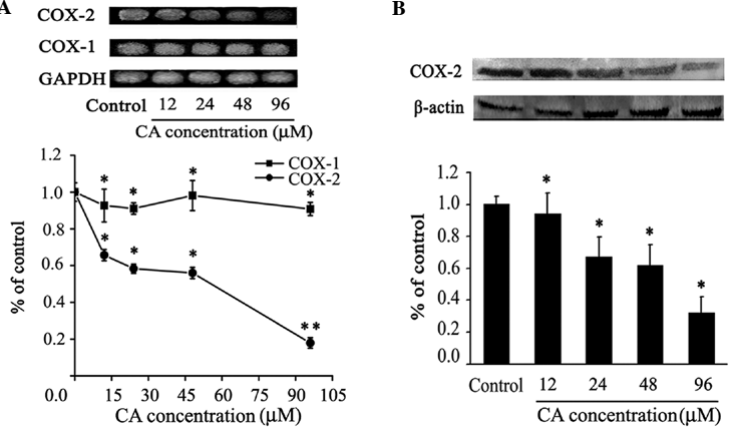
Effect of CA on the expression of COX-2 in Caco-2 cells. (A) Determination of COX-2, COX-1 and GAPDH mRNA by use of RT-PCR. The cells were incubated without or with CA (0 at 96 μM) for 24 h, and subsequently RNA was isolated and reverse transcribed; cDNA of COX-2, COX-1 and GAPDH were amplified by specific primers. The amplified cDNA products were quantified and the percentage of inhibition was determined. (B) The expression of protein COX-2 was assessed by western blot analysis. Levels of COX-2 (top) and β-actin (bottom) were quantified and the percentage of inhibition was determined. The values represent means ± SD of three experiments. (^*^P<0.05, ^**^P<0.01 vs. control).

COX-2 has been strongly implicated in intestinal and colon tumor growth (9,31).The effect of CA on the levels of COX-1 and COX-2 expression in Caco-2 cells was studied by RT-PCR and western blot analysis. Amplification by PCR of Caco-2 cDNA with COX-1, COX-2 and GAPDH primers produced bands of 0.3, 0.4 and 0.6 kb, respectively, as expected for the respective mRNAs. As shown in Fig. 7A, CA treatment had no effect on the expression of COX-1 mRNA (a constitutively expressed gene responsible for housekeeping PG biosynthesis), whereas it significantly downregulated COX-2 mRNA expression.

Fig. 7B shows that Caco-2 cells express detectable levels of COX-2 protein. However, the expression of COX-2 was reduced after 24-h CA treatment, inhibiting approximately by 50% its expression with 24–48 μM of CA. A strong inhibition effect on COX-2 expression at both protein and mRNA levels were observed assaying 96 μM of CA. These findings suggest that CA could act as a COX-2 inhibitor.

## Discussion

### Rosmarinus officinalis

L. is a medicinal plant with an elevated content of anti-oxidant polyphenols as CA and RA. In the present study, we demonstrated in human CRC cell lines the antiproliferative and apoptotic effects of CA as well as its inhibitory effect on other hallmarks of tumor progression such as migration and adhesion.

Although RA and CA were capable of suppressing cell growth to different degree; CA was the most active polyphenol since its antiproliferative effect was accompanied by substantial dose-dependent cytotoxicity in the three CRC lines examined: LoVo, HT29 and Caco-2 with different genetic backgrounds. In Caco-2 cell line, we showed that CA at IC_50_ (92.1 μM) was associated with induction of apoptosis, as evidenced by the translocation of phosphatidylserine in the plasma membrane (Fig. 3A), chromatin condensation, and loss of normal nuclear architecture (Fig. 3B). Other authors have described that CA inhibits DNA synthesis on Caco-2 cells at 23 μM using [^3^H]thymidine incorporation assay, and transient cell cycle arrest in G2/M phase with 50 μM CA (25). The antiproliferative effect of CA (2.5–10 μM) was also reported on HL-60 and U937 human myeloid leukemia cells attributed to inhibition of cell cycle progression with a transient blockage in the G1 phase (14), while another study with HL-60 cell reported that a high dose of CA (100 μM) induces apoptosis associated with activation of caspase-9 and -3 (26). We found that Caco-2 cells are arrested in G2/M after incubation with a RE containing approximately 30 μM of CA (data not shown).

Tumor invasion requires degradation of basement membranes (BM), which separates the epithelial and mesenchymal cell compartments, and is composed of macromolecules such as collagen, laminin, and heparan sulfate. A number of proteolytic enzymes, including MMPs and serine proteases, are involved in the degradation of the BM. In particular, activated MMP-2 and MMP-9 play an important role in BM degradation because of their ability to cleave collagen. Among serine proteases, the urokinase type plasminogen activator (uPA), which triggers a proteolysis cascade by accelerating the conversion of plasminogen into plasmin, is important for tumor invasiveness and metastasis and its expression is increased in solid tumors. Plasmin can degrade fibrin, fibronectin, proteoglycans, and laminin found in the tumor-surrounding matrix, activates collagenases and indirectly degrades collagens (27). We determined that CA has an inhibitory effect on Caco-2 cell adhesion to type I collagen and fibronectin surfaces (Fig. 4A and B). In addition, we documented the inhibition of spreading and pseudopodial extension of cells pre-treated with CA IC_50_ (Fig. 4C). Nevertheless, additional experiments will be required to identify the precise underlying mechanism.

Tumor cell migration is necessary at the first steps of the metastatic cascade, when cancer cells leave the primary tumor and gain access to the circulation, and also when malignant cells extravasate into the parenchyma of the secondary site. Tumor cells have a motile response to many agents, including host-derived motility and growth factors, ECM components, and tumor-secreted factors. In this study, we demonstrated by the wound healing assay that CA can inhibit Caco-2 cell migration in a dose-dependent manner (Fig. 5). Migration inhibition (50%) was reached at 48 μM of CA, approximately half the CA IC_50_ dose. Moreover, we found that at identical concentrations CA decreases the activity of important ECM-degrading proteases, as uPA, MMP-9 and MMP-2 which are closely associated with tumor progression. Our results show that CA treatment after 24 h decreased Caco-2 conditioned media uPA activity and MMP-9 and MMP-2.

Natural compounds as potential inhibitors of key cell signaling pathways such as COX-2 have gained much attention and therapeutic regimens with either the compounds alone or in combination with existing chemotherapeutic agents have been investigated (28). The expression of COX-2 is involved in tumor promotion during CRC progression (23,29-31). We have determined that CA downregulates the expression of COX-2 in Caco-2 cells at both mRNA and protein levels (Fig. 7A and B). Interestingly, COX-1 mRNA level was not affected by CA. The expression of COX-2 protein was reduced about 2-3-fold after the treatment with the IC_50_ dose of CA. Therefore, the growth inhibitory effect of CA may be mediated through a mechanism that probably involves inhibition of the COX-2 pathway.

The clinical importance of this effect lies in the fact that CA could offer therapeutic benefits of non-steroidal anti-inflammatory drugs (NSAIDs) (30) with reduced toxicity to the gastrointestinal mucosa.

In conclusion, we have demonstrated that CA inhibited Caco-2 cell growth by inducing apoptosis and reducing adhesion, migration and proteolytic enzyme activities, most probably by downregulation of COX-2 mRNA expression. Collectively, our results suggest that CA might modulate different targets involved in proliferation and apoptotic pathways. These findings indicate that CA may serve as chemopreventive and/or chemotherapeutic agent against colorectal cancer progress.

## Figures and Tables

**Table I:** IC_50_ of CA on CRC cell lines.

	Cell lines		
	
Carnosic acid	Caco-2	HT29	LoVo
IC_50_ (μM)	92.1+6.4	48.5+8.2	26.4+2.7
